# High impact of quadrivalent human papillomavirus vaccine across racial/ethnic groups: National Health and Nutrition Examination Survey, 2003–2006 and 2015–2018

**DOI:** 10.1080/21645515.2024.2308378

**Published:** 2024-02-19

**Authors:** Ruth Stefanos, Rayleen M. Lewis, Troy D. Querec, Julia W. Gargano, Elizabeth R. Unger, Lauri E. Markowitz

**Affiliations:** a Division of Viral Diseases, National Center for Immunization and Respiratory Diseases, Centers for Disease Control and Prevention, Atlanta, GA, USA; b Epidemic Intelligence Service, Centers for Disease Control and Prevention, Atlanta, GA, USA; c Division of High-Consequence Pathogens and Pathology, National Center for Emerging and Zoonotic Infectious Diseases, Centers for Disease Control and Prevention, Atlanta, GA, USA

**Keywords:** HPV vaccine, cervical cancer, race/ethnicity, National Health and Nutrition Examination Survey, adolescents and young adult females

## Abstract

Human papillomavirus (HPV) causes cervical as well as other cancers. Racial and ethnic disparities in cervical cancer incidence and mortality in the United States are well documented. HPV vaccination has been recommended in the United States since 2006 and is expected to prevent HPV-attributable cancers in all racial/ethnic groups. Quadrivalent HPV vaccine-type (HPV6/11/16/18) and nonvaccine-type cervicovaginal HPV prevalences were estimated from National Health and Nutrition Examination Surveys in 2015–2018 (vaccine era) and 2003–2006 (prevaccine era) data. Prevalence ratios comparing 2015–2018 to 2003–2006 were calculated among sexually experienced Non-Hispanic White (NHW), Non-Hispanic Black (NHB), and Mexican American (MA) females aged 14–24 years. Quadrivalent HPV vaccine-type prevalence declined 82% (CI: 60%–92%) among NHW, 86% (CI: 64%–95%) among NHB, and 100% among MA females, forecasting future reductions in cervical cancer across racial/ethnic groups.

## Introduction

Human papillomavirus (HPV) is the most common sexually transmitted infection;^1^ high-risk types can cause cervical cancer and other anogenital and oropharyngeal cancers. Low-risk types can cause anogenital warts.^2,3^ Underlying historical and current societal factors in the United States have resulted in cancer health disparities by racial/ethnic group, including disparities in cervical cancer incidence and outcomes that continue to persist.^4–7^ In 2016–2020, the age-adjusted cervical cancer incidence and mortality rates were higher in Non-Hispanic Black and Hispanic females compared to Non-Hispanic White females.^8^

The Advisory Committee on Immunization Practices recommends routine HPV vaccination for females at age 11–12 years with catch-up through age 26 years.^9^ Quadrivalent HPV vaccine (4vHPV) targeting HPV6/11/16/18 was licensed in 2006; 9-valent HPV vaccine (9vHPV), which targets an additional 5 types, HPV31/33/45/52/58, was licensed in 2014. Most HPV vaccine administered in the United States through 2015 was 4vHPV; since the end of 2016, only 9vHPV has been available in the United States.^10^

Cervicovaginal HPV prevalence monitoring in the National Health and Nutrition Examination Survey (NHANES) has demonstrated HPV vaccine impact in the United States. In 2015–2018, 4vHPV-type prevalence among sexually experienced females aged 14–24 years was 85% lower compared to 2003–2006.^11^ Given the racial/ethnic group disparities in cervical cancer, ongoing monitoring is important to evaluate continued impact of HPV vaccination by race/ethnicity. This study provides estimates of vaccine impact by race/ethnicity on 4vHPV-type prevalence and evaluates changes in prevalence of HPV31/33/45/52/58 using 2015–2018 data, the most recent NHANES data.

## Methods

NHANES is a nationally representative survey conducted by the National Center for Health Statistics (NCHS) at the Centers for Disease Control and Prevention (CDC).^12^ NHANES uses a complex multi-stage cluster sampling survey design. Specific subgroups are oversampled to produce reliable estimates. NHANES is reviewed and approved by the NCHS Ethics Review Board (Protocol Numbers: 98–12, 2005–06, 2011–17, 2018–01).

NHANES staff obtained informed consents for home interviews, health exams at the mobile examination centers (MEC) which include health measurements and tests, and specimen storage for future studies. During interviews, staff collected demographic information (e.g., age, race, ethnicity) and HPV vaccination history from participants. Participants self-collected cervicovaginal samples and reported sexual behavior information via audio computer-assisted self-interviews at a MEC. The CDC tested samples for 37 HPV types (6, 11, 16, 18, 26, 31, 33, 35, 39, 40, 42, 45, 51, 52[XR], 53, 54, 55, 56, 58, 59, 61, 62, 64, 66, 67, 68, 69, 70, 71, 72, 73, 81, 82, 83, 84, 89, and IS39) using the Research Use Only Linear Array HPV Genotyping Test (Roche Molecular Diagnostics), as described previously.^13^

As in a previous report,^11^ we restricted this analysis to female participants aged 14–24 years, who were sexually experienced as defined by NHANES (ever having had anal, oral, or vaginal sex) and had adequate HPV typing results in 2015–2018 (vaccine era) or 2003–2006 (prevaccine era). As this report focuses on analyses by race/ethnicity, we further restricted the analysis to female participants who were Non-Hispanic White (NHW), Non-Hispanic Black (NHB), or Mexican American (MA) (*n* = 1,480). Other racial/ethnic groups were not analyzed because they were not adequately sampled in 2003–2006 to produce reliable estimates.

All analyses were stratified by race/ethnicity (NHW, NHB, MA). We analyzed age at first sex, number of lifetime sex partners (≥3 lifetime partners vs <3 lifetime partners), self-reported vaccination status (≥1 vaccine dose), age at receipt of first HPV vaccine dose, and HPV prevalence by era (2015–2018 vs 2003–2006). We used Wald chi-square tests to determine differences in number of lifetime sex partners between 2015–2018 and 2003–2006 and defined statistical significance as *p* < .05. We estimated prevalence of 4vHPV, HPV31/33/45/52/58 (additionally targeted by 9vHPV), and non-9vHPV types. We determined the prevalence of non-9vHPV types to assess comparability of exposure to HPV between the two time periods. We calculated prevalence ratios (PRs) and 95% confidence intervals (CIs) for 2015–2018 compared to 2003–2006. Vaccine impact (percent decline in prevalence) was calculated as (1 – PR) x 100. Statistical significance for a PR was defined as a 95% CI that does not include 1. Relative standard errors (RSEs) were calculated for prevalence estimates, and RSEs > 30% were noted and considered unstable. All estimates were weighted to account for nonresponse and the complex survey design. Analyses were conducted in SAS version 9.4 (SAS Institute, Cary, NC) and SUDAAN, version 11.0 (RTI International, Research Triangle Park, NC).

## Results

Among sexually experienced females aged 14–24 years, the median age of first sex was similar between the prevaccine and vaccine eras in all three racial/ethnic groups (Table 1). The percentage reporting ≥3 lifetime partners was similar in 2015–2018 compared to 2003–2006 among NHW females (60.5% vs 60.4%) and NHB females (69.8% vs 67.8%), but was different by time period for MA females (54.8% vs 41.8%). In 2015–2018, HPV vaccination coverage was 60.9% in NHW, 57.2% in NHB, and 57.0% in MA females. Among those vaccinated, the median age at receipt of the first HPV vaccine dose was 14.2 years for NHW, 13.2 years for NHB, and 13.2 years for MA females.

**Table 1.** Characteristics of sexually experienced females aged 14–24 years by race/ethnicity, NHANES 2003–2006 & 2015–2018.

	Non-Hispanic White		Non-Hispanic Black		Mexican American	
	2003–2006	2015–2018	2003–2006	2015–2018	2003–2006	2015–2018
	n=371	n=196	n=358	n=132	n=292	n=131
Age first sex (yr), median (IQR)	15.3 (14.0–16.9)	15.7 (14.5–17.1)	14.9 (13.5–16.3)	15.4 (14.2–16.7)	15.8 (14.3–17.5)	15.4 (14.4–16.5)
≥3 lifetime sex partners, % (95% CI)	60.4 (56.4–64.4)	60.5 (54.6–66.2)	67.8 (63.3–71.9)	69.8 (61.4–77.1)	41.8 (35.3–48.5)	54.8 (45.5–63.8)*
≥1 HPV vaccine dose, % (95% CI)	-	60.9 (53.5–67.9)	-	57.2 (49.1–65.0)	-	57.0 (47.1–66.4)
Age first dose (yr), median (IQR)	-	14.2 (12.3–15.8)	-	13.2 (11.7–15.4)	-	13.2 (11.9–14.8)

IQR - Interquartile range; NHANES - National Health and Nutrition Examination Survey. Notes: All estimates are weighted. 2003–2006 is prevaccine era. * *p* < .05 based on Wald chi-square test comparing vaccine era to prevaccine era; - : not collected in those years since not relevant in prevaccine era.

4vHPV-type prevalence declined from 20.1% to 3.6% among NHW females (PR: 0.18; 95% CI: 0.08–0.40), from 21.4% to 2.9% among NHB females (PR: 0.14; 95% CI: 0.05–0.36), and from 11.5% to 0% among MA females (PR: undefined) (Table 2). Analysis of other HPV-type categories showed that HPV31/33/45/52/58 prevalence declined from 13.3% to 6.5% in NHW females (PR: 0.49; 95% CI: 0.25–0.94). Nonstatistically significant declines in HPV31/33/45/52/58 prevalence were also observed in NHB females, 24.7% to 18.9% (PR: 0.77; 95% CI: 0.45–1.29), and in MA females, 12.7% to 5.7% (PR: 0.45; 95% CI: 0.17–1.23). Non-9vHPV–type prevalences were similar in both eras for all racial/ethnic groups (range of PRs: 0.92–0.96).

**Table 2.** Cervicovaginal HPV prevalence among sexually experienced females aged 14–24 years by race/ethnicity, NHANES 2003–2006 & 2015–2018.

	Non-Hispanic White				Non-Hispanic Black				Mexican American			
	2003–2006	2015–2018	Comparison of 2015–2018 with 2003–2006		2003–2006	2015–2018	Comparison of 2015–2018 with 2003–2006		2003–2006	2015–2018	Comparison of 2015–2018 with 2003–2006	
	*n* = 371	*n* = 196			*n* = 358	*n* = 132			*n* = 292	*n* = 131		
HPV Types	% (95% CI)	% (95% CI)	PR (95% CI)	% Decline (95% CI)	% (95% CI)	% (95% CI)	PR (95% CI)	% Decline (95% CI)	% (95% CI)	% (95% CI)	PR (95% CI)	% Decline (95% CI)
6/11/16/18	20.1 (16.9–23.8)	3.6 (1.6–8.0)^†^	0.18 (0.08–0.40)*	82 (60–92)	21.4 (15.5–28.9)	2.9 (1.1–7.2)^†^	0.14 (0.05–0.36)*	86 (64–95)	11.5 (5.9–21.2)^†^	0^a^	N/A^a^	100%^a^
31/33/45/52/58	13.3 (9.2–18.9)	6.5 (3.7–11.3)	0.49 (0.25–0.94)*	51 (6–75)	24.7 (18.2–32.7)	18.9 (11.9–28.9)	0.77 (0.45–1.29)	23 (−29–55)	12.7 (7.6–20.4)	5.7 (2.3–13.5)^†^	0.45 (0.17–1.23)	55 (−23–83)
Non-9-valent	46.4 (41.6–51.2)	42.7 (34.3–51.6)	0.92 (0.74–1.15)	8 (−15–26)	66.6 (60.2–72.4)	63.9 (54.1–72.6)	0.96 (0.81–1.14)	4 (−14–19)	43.3 (32.1–55.1)	41.0 (31.2–51.6)	0.95 (0.66–1.36)	5 (−36–34)

PR - Prevalence ratio; NHANES - National Health and Nutrition Examination Survey.

All estimates are weighted. 2003–2006 is prevaccine era. *Confidence interval does not include 1. ^†^Relative standard error >30%

^a^The prevalence estimate was 0%. The prevalence ratio for this estimate is undefined and no confidence intervals were calculated.

## Discussion

This report demonstrates over 80% declines in 4vHPV-type prevalence among NHW, NHB, and MA females aged 14–24 years in 2015–2018 compared to 2003–2006. This expands on data in a previous report for this survey, which showed that 4vHPV-type prevalence declined by 85% among females overall in this age group.^11^ As in that study, we restricted this analysis to sexually experienced females because a prior NHANES analysis showed that the percentage of females aged 14–19 years reporting ever having sex decreased.^14^ This restriction made it more likely that declines in 4vHPV-type prevalences were attributable to vaccination and not to changes in sexual behavior. Furthermore, we found that prevalences of HPV types not targeted by any vaccine were stable between the two time periods, showing similar exposure to HPV in all racial/ethnic groups.

This analysis updates previous estimates by race/ethnicity from 2013–2016.^15^ In that report, prevalence of 4vHPV types declined among females aged 14–19 years in all racial/ethnic groups analyzed (NHW, NHB, MA) in 2013–2016 compared to 2003–2006, but smaller declines were observed in NHB (70%) than the other groups (NHW, 86%; MA, 84%). 4vHPV-type prevalence declined among females aged 20–24 years in NHW (72%) and NHB (57%) females as well.^15^ In our updated analysis, we found declines in 4vHPV-type prevalence that were statistically significant and high in all three racial/ethnic groups: declines of 82% in NHW, 86% in NHB, and 100% in MA females aged 14–24 years. We combined females aged 14–19 and 20–24 years because of low 4vHPV-type prevalence in 2015–2018; therefore, our results cannot be directly compared with the previous analysis.

Prevaccine era HPV31/33/45/52/58 prevalences were not the same in all racial/ethnic groups. HPV31/33/45/52/58 prevalence was almost two-fold higher in NHB than NHW or MA females. We found significant decreases in prevalence of HPV31/33/45/52/58, types targeted by 9vHPV, among NHW females (51%); decreases among NHB females (23%) and MA females (55%) were also observed, although these decreases were not statistically significant, likely due to the smaller number of participants in those groups.

Almost all HPV vaccine administered through the end of 2015 in the United States was 4vHPV. Since the end of 2016, 9vHPV has been the only HPV vaccine marketed in the United States.^10^ Information on vaccination with 9vHPV was collected in interviews conducted during the 2017–2018 NHANES; prior NHANES surveys did not distinguish between 4vHPV and 9vHPV products. However, most females across all racial/ethnic groups surveyed in 2015–2018 reported receiving their first dose earlier than 2015; three quarters of vaccinated NHW and NHB females reported receiving their first dose when they were aged <16 years, and three quarters of vaccinated MA females reported receiving their first dose when they were aged <15 years. Therefore, a minority of females could have received 9vHPV. It is possible that declines in the additional types targeted by 9vHPV in this analysis were due to a combination of cross protection from 4vHPV and some direct protection from the use of 9vHPV. While results vary in different studies, some cross protection has been reported for bivalent HPV vaccine; more limited cross protection has been reported for 4vHPV.^16^ Because of the transition from 4vHPV to 9vHPV during 2015–2016 in the United States, future NHANES surveys will include more participants who had the opportunity to be vaccinated with 9vHPV. 9vHPV impact will be better described by these future NHANES surveys.

The declines in 4vHPV-type prevalence across racial/ethnic groups are consistent with similar HPV vaccination coverage reported in NHANES by race/ethnicity. In this analysis, 57% of NHB and MA females and 61% of NHW females self-reported receiving ≥1 HPV vaccine dose. In a younger adolescent sample, the National Immunization Survey-Teen (NIS-Teen), which relies on provider report of vaccination history and measures coverage in adolescents aged 13–17 years, has consistently found coverage of ≥1 HPV vaccine dose is higher among some racial/ethnic groups compared with NHW. For example, in 2018, ≥1 HPV vaccine dose coverage among NHB adolescents was 72.8% compared to 63.5% among NHW adolescents.^17^ Differences in vaccination coverage in NHANES and NIS-Teen may be due to methodologic differences noted above. The large declines in vaccine-type prevalence relative to the vaccination coverage is likely due to herd protection from female vaccination as well as from male vaccination, recommended in 2011. Herd protection from HPV vaccination programs has been observed in the United States and in other countries.^18^ While dramatic declines in 4vHPV-type prevalence have been observed in the United States, HPV vaccination coverage is still below levels reached for other adolescent vaccines.^19^ Efforts are needed to increase HPV vaccination across all racial/ethnic groups and to reach those who missed vaccination due to the COVID-19 pandemic.

This study is subject to limitations. Some subgroup estimates had limited precision, especially in 2015–2018 when vaccine impact resulted in few detections of 4vHPV types. We could not present data on all racial/ethnic groups separately; Asian and other Hispanic groups were oversampled in 2015–2018 but were not adequately sampled in 2003–2006 for comparison. Finally, due to self-report of vaccine history, misclassification of vaccination status might be present.

## Conclusion

Within 12 years of HPV vaccine introduction, the declines in cervicovaginal 4vHPV-type prevalence demonstrate high impact of the vaccination program in all racial/ethnic groups evaluated. Continued declines (>80%) across racial/ethnic groups in young females support vaccination as a tool to help overcome racial/ethnic disparities in cervical cancer. Ongoing monitoring is needed to evaluate the 9vHPV impact by race/ethnicity.

## Data Availability

The data that support the findings of this study are a combination of publicly available and restricted data. Please see the NHANES website for more information: https://www.cdc.gov/nchs/nhanes/index.htm.

## Prior presentations

This work was included in a poster at EUROGIN (February 2023, Spain, Abstract # 4836) and presented at CDC’s Epidemic Intelligence Service (EIS) Conference (April 2023, Atlanta, GA).
